# High-resolution mapping of transcription factor binding sites on native chromatin

**DOI:** 10.1186/1756-8935-6-S1-P114

**Published:** 2013-04-08

**Authors:** Sivakanthan Kasinathan, Steven Henikoff

**Affiliations:** 1 Basic Sciences Division, Fred Hutchinson Cancer Research Center, Seattle, WA 98109, USA; 2 Medical Scientist Training Program and Molecular and Cellular Biology Graduate Program, University of Washington, Seattle, WA 98195, USA; 3 Howard Hughes Medical Institute, Seattle, WA 98109, USA

## 

Sequence-specific DNA-binding proteins including transcription factors (TFs) are key determinants of gene regulation and chromatin architecture. Formaldehyde cross-linking and sonication followed by chromatin immunoprecipitation and sequencing (X-ChIP-seq) is the most widely used technique for genome-wide profiling of protein binding sites. However, there are many issues associated with X-ChIP including low resolution and poor specificity and sensitivity. Here, we implement native (i.e., without cross-linking) ChIP of micrococcal nuclease-digested chromatin followed by paired-end sequencing (N-ChIP-seq) for mapping binding sites of the structurally distinct budding yeast TFs Abf1 and Reb1. N-ChIP-seq reproducibly recovers Abf1 and Reb1 binding sites with higher specificity and sensitivity than other profiling methods and identifies both previously characterized and novel sites. Altering N-ChIP-seq conditions allows flexibility in modulating specificity and sensitivity of binding site detection. Further, unlike X-ChIP methods, N-ChIP-seq is not biased toward identifying sites in accessible chromatin. Taken together, these results suggest that N-ChIP-seq outperforms current X-ChIP methodologies for genome-wide profiling of TF binding sites.

